# Management of Allergic Rhinitis in the United Arab Emirates: Expert Consensus Recommendations on Allergen Immunotherapy

**DOI:** 10.7759/cureus.65260

**Published:** 2024-07-24

**Authors:** Fares Zaitoun, Hamad Al Hameli, Marilyn Karam, Ravi Gutta, Eike Wustenberg, Tushar Arora, Mohamed Abuzakouk

**Affiliations:** 1 Allergy and Immunology, Clemenceau Medical Center Hospital, Dubai, ARE; 2 Allergy and Immunology, Cleveland Clinic, Abu Dhabi, ARE; 3 Allergy and Immunology, Saudi German Hospital, Dubai, ARE; 4 Allergy and Immunology, Mediclinic City Hospital, Dubai, ARE; 5 Otorhinolaryngology and Allergy, Dresden University, Hamburg, DEU; 6 Medical Affairs, ALK-Abelló, Copenhagen, DNK; 7 Medical Affairs, Biologix FZCO, Dubai, ARE

**Keywords:** united arab emirates, sublingual immunotherapy, subcutaneous immunotherapy, consensus, allergen immunotherapy, allergic rhinitis

## Abstract

Allergic rhinitis (AR) is a chronic inflammatory condition of the upper airways caused by a type I hypersensitivity reaction triggered by environmental allergens. AR is associated with significant morbidity and affects patients’ quality of life, emotional well-being, productivity, and cognitive functioning. As AR prevalence and morbidity have increased significantly worldwide, similar observations have been noted in the United Arab Emirates (UAE) with AR becoming a potential public health issue. Management of AR in the UAE is mainly provided by non-allergy specialists relying on first-line treatments such as intranasal steroids and antihistamines, with often suboptimal and short-term efficacy. Allergen Immunotherapy (AIT) is the only currently available disease-modifying treatment option in the form of either subcutaneous or sublingual allergen immunotherapy that has been proven to have long-term benefits. This article aims to provide recommendations regarding the use of AIT for managing AR in the UAE, considering both the current landscape in the Emirati healthcare system and local experience.

## Introduction

Allergic rhinitis (AR) is a chronic inflammatory condition of the upper airways that affects the nasal mucosa and may often involve lower airway inflammation. It is caused by a type I hypersensitivity response, which occurs when sensitized individuals are exposed to specific inhaled allergens [1]. The prevalence of AR has been steadily increasing over the past three decades, affecting up to 30% of the population worldwide [2-5].

Rapid urbanization and economic development in the United Arab Emirates (UAE) have significantly altered the environmental landscape [6,7]. This includes a surge in population, urbanization, industrial activity, construction projects, and increased vehicle use, all leading to increased burning of fossil fuels and worsening outdoor air pollution [7]. These factors have likely and collectively contributed to deteriorating ambient air quality, particularly in major cities. Studies have shown that concentrations of fine particulate matter (PM) 2.5 in the UAE frequently exceed international standards and can be more than eight times higher than what is set by the World Health Organization (WHO) [8,9]. This rise in air pollution is a major public health concern and likely contributes to the high prevalence and morbidity of AR observed in the UAE [6,10]. 

AR is associated with a significant reduction in quality of life (QoL), disturbed sleep, learning difficulties, school and work performance deterioration, and social functioning, highlighting AR as a substantial economic burden and a critical worldwide health issue [5,11-14]. In the UAE, data from a large cross-sectional study conducted between November 2007 and January 2008 demonstrated that the standardized prevalence of concomitant asthma and AR was 7.3% [6]. In addition, people with AR had a three times higher risk of developing asthma than subjects without AR (23.8 and 7.5%, respectively) [6]. Sneezing, rhinorrhea, nasal itching, and congestion are all manifestations of AR. Outdoor allergens, particularly trees, grass, and weed pollen induce seasonal AR symptoms. Allergens found in house dust mites (HDM), animal dander, and molds cause symptoms of perennial AR (PAR) throughout the year [15-17].

Antihistamines and intranasal corticosteroids (INCS) are commonly used pharmacological options; however, they only temporarily reduce symptoms, with the possibility that many of those patients may not experience adequate and long-lasting symptom improvement [15,17-19]. Allergen immunotherapy (AIT) is the only currently available disease-modifying treatment option for patients with AR [20]. The treatment principle of AIT is the administration of the allergen(s) to which the patient is clinically sensitive, in relatively high doses and on a frequent repetitive basis [21]. This eventually results in the induction of allergen-specific tolerance and the subsequent reduction of symptoms upon future exposure. In subcutaneous immunotherapy (SCIT), the sensitizing allergens are injected in increasing dosages, initially twice weekly, weekly, or on a less frequent build-up schedule depending on the product used, followed by maintenance doses at periodic monthly intervals to complete at least a three-year course [22]. The injections are given under medical supervision in the clinic, followed by a 20-30-minute observation period.

Sublingual immunotherapy (SLIT) products, which are provided in the form of tablets or drops, are placed under the tongue for immediate absorption [23]. Depending on the product, little or no up-dosing may be needed. The daily administration of SLIT is self-administered by the patient at home after receiving the first dose under supervision in the clinic. This home administration of SLIT is advantageous in regard to its ease and convenience. Upon completing the three to five years of treatment duration, the persistent long-term therapeutic benefits and immunologic tolerance have been well documented for both SCIT and SLIT. However, clinical improvement starts long before completion [24,25].

AIT acts through a multifaceted mechanism, including early desensitization, modulation of T and B cell responses with a shift in antibody isotypes, and regulation of inflammatory cell migration and mediator release [26]. Thus, AIT directly reduces allergic inflammation, diminishing symptom severity, disease burden, and medication needs [27].

Given the significant rise in the prevalence and morbidity of AR, this study offers practical recommendations for managing AR in the UAE, especially in regards to SLIT, considering both the current available resources in the UAE and local medical practices.

## Materials and methods

A study group of six experts discussed statements and recommendations related to the prevalence, causes, and management of AR in the UAE, including the use of AIT. This panel included representatives from the UAE's board-certified allergy/immunology specialists who handled AR patients in various healthcare settings and one expert from Germany.

The final expert statements and recommendations were formulated into two phases. The first was anonymous online voting using a predetermined online questionnaire. The questionnaire was created following a thorough literature review of up-to-date published data and recommendations for diagnosing and managing patients with AR. It was made using the online survey tool SurveyMonkey® and had 33 questions. A third-party organization, RAY-Contract Research Organization, Egypt, helped organize this phase by collecting the data and performing the analyses.

The second development phase began after the data analysis was completed on December 18, 2022. This phase consisted of a single in-person meeting held in Dubai, UAE, in which all panel group members participated in multiple discussions, shared their thoughts and insights, and endorsed the final recommendations.

## Results

Prevalence of AR in the UAE

The responses of the expert panel to the online questionnaire are given in Table 1.

**Table 1:** Responses of the expert panel to the study questionnaire in the first phase

Questions	Level of Agreement (%)
Globally the prevalence of Allergic Rhinitis is estimated between 10%-30%; accordingly, what is the estimated prevalence of Allergic Rhinitis in UAE?	
10-20%	33.33%
20-30%	50.00%
30-40%	0%
40-50%	16.67%
50-60%	0%
What are the main factors that may affect the prevalence of Allergic Rhinitis in the UAE? (Please select all that apply)	
Airborne allergens, such as pollen, animal dander, dust mites, and mold	100%
Pollution	83.33%
Genetic hereditary	50.00%
Seasonal variations	33.33%
Others (Dust, AC)	16.67%
Insect stings, such as from a bee	0%
How many newly diagnosed Allergic Rhinitis patients do you see in your clinic per year?	
80-100	0%
100-150	0%
Above 150	100%
What is the estimated percentage of Seasonal Allergic Rhinitis in the UAE?	
10-20%	50%
20-40%	50%
40-60%	0%
60-80%	0%
80-100%	0%
What is the estimated percentage of Perennial Allergic Rhinitis in the UAE?	
10-20%	16.67%
20-40%	33.33%
40-60%	0%
60-80%	33.33%
80-100%	16.67%
What are the most common predisposing factors for Allergic Rhinitis? (Select all that apply)	
Atopy	100%
Asthma	66.67%
Parental history of allergic disease	50%
Eczema	50%
Other allergic diseases	50%
Psychological stress	0%
How does Allergic Rhinitis affect the patient’s quality of life? (Please select all that apply)	
Sleep disorder	100%
impairments of work productivity and performance	100%
fatigue	100%
Mood changes	70%-80%
Cognitive function	66.67%
Do you recommend offering Allergen Immunotherapy (AIT) to prevent Allergic Rhinitis (primary prevention)?	
Yes	0%
No	100%
Do you recommend possibly prescribing more than one AIT at the same time?	
Yes	83.33%
No	16.67%
How do you evaluate the effectiveness of AIT? (Please select all that apply)	
Symptom scores	100%
Visual analogue scores	33.33%
Assessments of allergen sensitivity in the eye, nose, or skin	16.67%
Symptom and mediation scores and utilization and prevention of complications	16.67%
Serum levels of IgE and IgG antibodies	0%
How do you assess the patient’s Quality of Life with AIT treatment? (Please select all that apply)	
Improvement in sleeping	100%
Less fatigue	100%
Better mood status	100%
Better work productivity and performance	100%
Feeling well	83.33%
Improvement in cognitive function	66.67%
When do you decide to start AIT for the management of AR patients?	
According to the patient’s preferences and desire for a cure	100%
Failure of conventional therapy for 3-12 months	100%
Combined with conventional treatment options	33.33%
Upon diagnosis of Allergic Rhinitis, what is your 1st line of treatment? (Please select all that apply)	
Corticosteroid nasal spray	100%
Oral antihistamines	50%
Oral decongestant	16.67%
Nasal spray	16.67%
Oral or Nasal antihistamines	16.67%
SLIT	0%
Based on your experience, how do you select the Allergic Rhinitis patient who is eligible for AIT? And what are these eligibility criteria? (Please select all that apply)	
Accurate diagnosis with a medical history of allergic rhinitis/ conjunctivitis and/or allergic asthma	100%
Poor symptom reduction despite adequate pharmacotherapy	100%
Shared decision-making considering the wishes of the patient (and the caregiver), is an essential part of the indication.	100%
Affordable cost for the patient	66.67%
Change in natural allergy history	16.67%
mHealth technologies, such as the MASK-air allergy app, can be of relevant importance for the selection of patients	16.67%
Prolonged need for pharmacotherapy needed for control of symptoms	16.67%
What comorbidities should be considered before prescribing AIT?	
Poorly controlled Asthma	100%
Cardiovascular disease	50%
Autoimmune disease	16.67%
Surgical co-factors and non-allergic factors	16.67%
Diabetes Mellitus	0%
Are there lower age limits for initiating AIT?	
Yes	66.67%
No	33.33%
For how long should an AIT be prescribed (as a minimum duration)?	
At least 1 year	0%
2 years	0%
3 years	83.33%
5 years	16.67%
Do you recommend prescribing other medications in combination with AIT treatments?	
Yes	100%
No	0%
In your opinion, what is the treatment duration for evaluating the efficacy of AIT?	
12-14 weeks	0%
4-6 months	16.67%
6-9 months	16.67%
9-12 months	16.67%
1 year	50%
2 years	0%
3 years	0%
When do you recommend the initial follow-up visit for patients started on AIT?	
4-8 weeks	100%
12-14 weeks	0%
4-6 months	0%
6-9 months	0%
9-12 months	0%
1 year	0%
What is the approach you use to assess the efficacy of AIT?	
Symptom Scores	100%
Medication scores	50%
Visual Analogue Scores	33.33%
Assessments of allergen sensitivity in the eye, nose, or skin	16.67%
Serum levels of IgE and IgG antibodies	0%
Based on your experience, in case of improvement, what is your recommendation?	
Continue for 3 years If a patient is improving, will continue for the full duration of 3 to 5 years Complete 3 years course	66.67%
Reduce the dose	33.33%
Discontinuation of the treatment	0%
Switching to another treatment	0%
Based on your experience, in case of little or no improvement, what is your recommendation? (Please select all that apply)	
Concomitant use with another anti-allergic medication	50%
Switching to another treatment	33.33%
Increase the treatment duration	33.33%
Increase the dose	16.67%
Reevaluate allergen sensitizations, exposure, route of immunotherapy, need for the subcutaneous treatment	16.67%
Do you think AIT is more cost-effective than other treatments in the long term in UAE?	
Yes	100%
No	0%
Do you face any rejection from your patient when prescribing AITs?	
Yes	100%
No	0%
If yes, do you inform the patient before prescribing about the treatment benefits, procedure, and duration of treatment?	
Yes	100%
No	0%
Do you face any rejections from insurance companies when prescribing AIT?	
Yes	66.67%
No	33.33%
What is the percentage of patients that stopped AIT without medical consultation?	
>10%	0%
10-20%	50%
20-30%	50%
<30%	0%
What are the main reasons for patients’ non-adherence to AIT?	
Lack of insurance coverage	100%
Lack of tangible efficacy in the early stages of treatment	66.67%
Long term treatment	66.67%
Tolerability/side effects	66.67%
Cost	33.33%
Do you face compliance difficulties with patients on AIT?	
Yes	83.33%
No	16.67%
What are the patients’ criteria for suitability for AIT (SLIT or SQIT)?	
Accurate diagnosis with a medical history of allergic rhinitis/ conjunctivitis and/or allergic asthma	100%
Poor symptom reduction despite adequate pharmacotherapy	100%
Shared decision-making considering the wishes of the patient (and the caregiver) is an essential part of the indication	83.33%
Affordable cost for the patient	66.67%
What are the expected benefits of Sublingual Immunotherapy (SLIT)?	
Easy route of administration	100%
Effective treatment	100%
High safety profile	100%
Low adverse event profile	83.33%
What are the guidelines that you follow in treating Allergic Rhinitis patients?	
AAAAI & ACAAI updated guidelines	83.33%
ARIA guidelines	66.67%
EAACI guidelines	66.67%
BSACI guidelines	33.33%
WAO guidelines	33.33%

AR affects 10-30% of the global population [28]; however, little is known about its incidence in the UAE and the rest of the Middle East [29]. The expert panel opined that due to UAE's climate, many patients with AR present with a mixed type of disease (combination of AR and non-AR (NAR), irritant or vasomotor rhinitis). However, AR is often unrecognized and undiagnosed in many individuals, so it is challenging to determine the true prevalence of AR in the UAE. The expert panelists estimated the prevalence of AR in the UAE to be in the range of 20-30%, with airborne allergens being the primary triggers. This is supported by the increasing indoor presence of pets and their dander, alongside detecting specific allergens like molds and pollen through IgE testing [10]. In addition to airborne allergens that directly trigger the IgE-mediated allergic reaction, ambient air pollution, sand dust, and other airborne irritants and triggers are considered a significant contributing factor in those with underlying genetic predisposition [10]. 

The number of newly diagnosed AR cases in the UAE is rising. The expert panel estimated that there will be more than 150 annual newly diagnosed AR cases per each expert clinic by 2025, with a seasonal percentage of AR of 10-20%. Moreover, the panel estimates that approximately 15% of patients with AR experience severe symptoms, 30% experience moderate symptoms, and the remaining 55% experience mild symptoms. Further, seasonal AR is observed from the end of March to mid-April and largely depends on the residential location within the UAE. For instance, residents of Al-Ain city are prone to present with grass and tree pollen allergies. In contrast, residents of Dubai and Abu Dhabi are prone to present with perennial allergens. A study by Alsowaidi et al. revealed that Al-Ain City, an inland desert oasis, is known for its diverse plant life, including some that produce anemophilous pollens, which are relevant allergens in the context of airborne AR triggers [29]. Desert regions with numerous buildings that generate fine dust include Dubai, Umm Al-Quwain, and Ajman. Ras Al-Khaimah has cement factories and ceramic industries. Fujairah contains mountains that are rich in plant life. These environmental variables are attributed to the natural history of AR in the region.

Predisposing factors for and symptoms of AR

Atopy was ranked first among predisposing factors of AR. Other common predisposing factors or associated conditions with AR include asthma, eczema, parental history of allergic disease, and the presence of other allergic diseases. However, assessing the burden of the disease is challenging. Despite the availability of AR disease-specific QOL tools, perceptions fluctuate constantly and may vary between and within individuals [30]. Chronic AR can lead to snoring or mouth breathing due to nasal obstruction, chronic sinusitis, headaches, sinus pressure, chronic cough, loss of taste or smell, or hearing dysfunction. These symptoms can cause interrupted sleep, diurnal fatigue, irritability, or sleepiness, which can negatively impact the emotional, physical, and social aspects of QoL [30,31]. 

The panel members agreed that fatigue, sleep disorders, work productivity, and performance impairments are the most crucial sequels of AR affecting QoL. Moreover, mood changes (including depression and anxiety) and their impact on cognitive function are also seen.

Allergen immunotherapy

The expert panel agreed that AIT may be offered to patients with insufficiently controlled AR symptoms and proof of clinically relevant allergen-specific sensitization by allergy skin testing or reliable serum-specific IgE testing. When AIT is being considered as a preventative treatment, they opined that it may be initiated at an earlier stage of the disease.

The panel stressed that the failure of conventional treatment is not a principal prerequisite to start AIT, i.e., patients who respond to conventional therapy can also be considered for AIT, especially when the patient desires an effective, long-term treatment strategy with the added advantage of preventing disease progression. These aspects are essential factors that should also be considered in clinical practice.

The panel recommended that patients consult with their physicians if contemplating stopping AIT, as previous experience of the experts had shown that around 20-30% of patients discontinued treatment without medical consultation. One of the leading reasons for premature stoppage, according to the panel, is the expectation of rapid benefit, whereas AIT typically requires several months to show a significant effect. In general, continual patient-physician communication during the AIT treatment course is important to manage expectations and address concerns, and the prescribing physician should periodically assess and review the original indications for AIT and determine if any developments, including contraindications, have arisen that would require the discontinuation of AIT.

Despite recognizing the potential benefits of AIT for individuals with AR across various severities, the panel discussed challenges that may hinder its wider adoption within the UAE. These challenges include limited awareness and understanding of AIT among patients and healthcare professionals, potential cost considerations and limitations in insurance coverage, individual patient preferences, and potential difficulties in adhering to long-term AIT therapy.

The panel agreed that while many patients are encouraged that AIT is a disease-modifying treatment and a potential cure, shortly into the course, they may question its efficacy and discontinue the AIT. Thus, setting expectations before starting AIT is crucial to avoid non-adherence and waste of resources.

The timing of AIT initiation is crucial for managing patients with AR. According to the panel, various aspects should be considered before making that decision, including “patient choice and preference,” which is essential to secure compliance. Therefore, comprehensive information about the different available treatment options for both SLIT (tablets or drops) and SCIT, including pros and cons, in addition to sound clinical evidence, along with availability, including registration status of the products, should be provided (Table 2).

**Table 2:** Pros and cons of SLIT versus SCIT SCIT: subcutaneous immunotherapy; SLIT: sublingual immunotherapy

SLIT		SCIT
Pros	At-home treatment (after the first dose in the clinic)	In the maintenance phase, a monthly injection
	Documented proof of efficacy	Single and mixed allergens
	Non-invasive	Documented proof of efficacy
	Registered and available in UAE	From Age 5
	SLIT drops (from Age 5)	
	SLIT tablet (from Age 16; Pediatric age approval expected soon)	
Cons	Daily intake	All Injections at the doctor’s office followed by a waiting period
	Only available for specific allergens	Frequent injections to achieve the maintenance phase
	Possible mild local reactions at the beginning of treatment	More potential for acute systemic reaction

Currently, the only commercially available AIT products covered by third-party payers in the UAE are Acarizax sublingual tablets and Staloral sublingual drops [32,33]. The specific product selection should be individualized based on the patient's allergen profile and treatment preferences.

Patients should be informed that perhaps due to AIT's mechanism of action in the case of SLIT tablets, mild-to-moderate transient local adverse reactions like oral itching and swelling may occur, and observed clinical benefits may not be perceived until several weeks after initiation.

In the case of SLIT tablets, only the initial dose is administered in the clinic; otherwise, SLIT is taken by the patient at home [34]. Therefore, the patient should receive thorough education about the treatment at the initial intake, and it should be confirmed that the patient is aware of the proper placement of the tablet. It may be helpful to let the patient take the first tablet in front of a mirror under observation by the physician.

Product Selection

For AIT, both SCIT and SLIT products are available for a variety of allergens commonly encountered in the UAE, such as house dust mites, regional pollens, and molds. The products are not directly comparable (differences in allergen content, adjuvants, application routes, clinical documentation, etc.), and thus major international guidelines recommend a product-specific evaluation [21,35]. The panel strongly recommends prioritizing allergen immunotherapy products with well-established clinical efficacy demonstrated through double-blinded controlled trials and, whenever available, supported by real-world evidence. Specifically, many clinical trials reported the efficacy of Acarizax [36,37]. Similarly, Staloral was also found to be an effective AIT option in sublingual drops [38]. Healthcare professionals are encouraged to consult current guidelines and resources to stay informed about the latest evidence regarding the efficacy of specific AIT products available in the UAE.

Whenever available, choosing products with proof of clinical efficacy in double-blinded placebo-controlled trials and preferably a corresponding marketing authorization is strictly recommended. Furthermore, SLIT tablets are widely documented in many large clinical trials, leading to corresponding marketing authorizations by major worldwide regulatory authorities such as the European medicines agency (EMA) or the Food and Drug Administration (FDA) [39,40]. In addition, SCIT products need to be injected in the clinic, followed by a 20-30-minute observation period to identify and manage an unlikely but possible immediate allergic reaction [21]. The first intake of SLIT products is also performed in the clinic, followed by a 30-minute waiting time. However, if the first intake is tolerated well, the patient can continue the treatment at home, which is much more convenient.

Use of AIT in Poly-Sensitized Patients

Many AR patients are poly-sensitized. There exists an established debate as to whether it is preferable or necessary to include in the AIT vaccine all or most of the allergens to which the patient is sensitized (United States approach) or simply a single allergen that appears to be the cause of the majority of the patient’s symptoms, which is the preferred treatment option in Europe [41].

In the case of using SLIT tablets, data exist for mono- and polysensitized patients. Regardless of their level of sensitivity, one notable study showed that the patient’s symptoms significantly improved after taking HDM SLIT tablets [42,43]. Similarly, in another study, patients with poly-sensitivity who received only grass pollen immunotherapy showed similar clinical improvement [44].

The first sequential and later simultaneous administration of two SLIT tablets appears well tolerated. One hundred and two adults allergic to grass and ragweed pollens were given both types of SLIT tablets in combination in an open-label six-week study to assess tolerability [45]. Two weeks of sequential administration of each tablet was followed by two weeks of administering both tablets simultaneously, five minutes apart. Giving one type of tablet in the morning and another in the evening is an alternative strategy.

Assessment of Efficacy of AIT

The expert panel agreed that the efficacy of the AIT can be assessed mainly by assessing symptom frequency and severity using symptom scores. Medication scores, visual analog scores, utilization and prevention of complications, and assessments of allergen sensitivity in the eye, nose, or skin can also be considered. Assessment of the patient’s QoL with AIT treatment is typically built upon improved sleep, less fatigue, better mood status, better work productivity and performance, improved cognitive function (especially in school-age children), and feeling well. The panel’s statements and recommendations on AR in the UAE and the use of AIT as a treatment option are depicted in Table 3.

**Table 3:** Statements and recommendations regarding AR in the UAE and the use of AIT as a treatment option AIT: allergen immunotherapy; AR: allergic rhinitis; QoL: quality of life; UAE: United Arab Emirates; SCIT: subcutaneous immunotherapy; SLIT: sublingual immunotherapy

Statements and Recommendations
The estimated prevalence of AR in the UAE is 20-30%.
Airborne allergens and the effect of ambient air pollution are mainly responsible for the high prevalence of AR in the UAE.
About 10-20% of patients with AR in the UAE have seasonal AR (SAR).
About 60-80% of patients with AR in the UAE have perennial AR (PAR).
Atopy is the most common predisposing factor for AR.
Fatigue, sleep disorders, and impairments of work productivity and performance are the most crucial sequels of AR on QoL. Mood changes (including depression and anxiety) and impact on cognitive function are typically observed too.
AIT should not be offered for the primary prevention of AR.
Patient choice should be an essential consideration when prescribing and initiating AIT.
The failure of conventional treatment is not a principal prerequisite to starting AIT, i.e., patients who respond to conventional therapy can also be considered for AIT.
Whenever available, AIT products with proven efficacy and marketing authorization should be used.
The management of patients’ expectations is essential to improve compliance, focusing on the mode of action of AIT, possible early local reaction in case of SLIT, and lack of early perceived benefits.
Treating patients with multiple allergens is possible with both SCIT and SLIT. Further research and expert guidance is needed to determine the most appropriate administration approach, including sequential administration in the case of SLIT.
Physicians should use validated symptom score scales as a primary tool for evaluating the effectiveness of AIT.
Physicians should assess the impact of AIT on patients’ quality of life by using either a quantitative or qualitative approach by exploring improvements in sleep, fatigue, mood, work/school productivity, and overall well-being during consultations.

Patient Profiles and Regimen for AIT

Upon diagnosis of the AR patients, the experts recommend corticosteroid nasal spray as the first-line treatment. In addition, the expert panel recommends considering saline irrigation as an add-on therapy for patients with persistent symptoms. While some studies suggest limited additional benefits, adding oral H1 antihistamines might offer further relief for some patients, particularly in the context of limited resources and expertise faced by non-allergy specialists [46,47]. The decision to add H1 antihistamines should be individualized based on patient response and tolerability. AIT is the only currently available disease-modifying treatment modality to prevent disease progression and develop new sensitizations [48]. The expert panel agreed that the selection of patients with allergic rhinitis eligible for AIT treatment should be based on an accurate diagnosis of allergic rhinitis/rhinoconjunctivitis and poor symptom control despite the use of standard medications and adequate pharmacotherapy, proven sensitization by prick test and/or sIgE, and shared decision-making considering the patient and/or caregiver preference. Consulting with the Allergy specialist is recommended if further diagnostic clarification, confirmation, or expert opinion is needed.

A patient’s ability to afford the cost is another crucial aspect to be discussed. Patients with coexisting asthma may also be treated with AIT. However, poorly controlled asthma is a main contraindication of prescribing AIT. Also, cardiovascular disease comorbidities, typically viewed as relative contraindications for AIT, especially for SCIT, should be considered. As there are significant differences between AIT products regarding contraindications, a careful review of the specific product characteristics is essential before prescribing. While SCIT is generally not recommended for children under five, other factors influencing eligibility for both SCIT and SLIT include uncontrolled asthma, severe immune system disorders, and pregnancy [49,50]. Consulting with an allergist is recommended for patients with complex medical conditions or who fall outside the typical age range for AIT initiation.

The experts agreed that AIT should be prescribed for at least 3 years, which may be extended to 5 years. The initiation of SLIT AIT should be succeeded by a follow-up visit, preferably 4 weeks later, to secure compliance. A minimum of one year is typically needed to evaluate the effectiveness of AIT for both SLIT and SCIT. If a patient experiences minimal or no improvement, a comprehensive re-evaluation of the diagnosis, treatment adherence, and overall treatment plan is crucial. Consulting with the Allergy specialist would be beneficial if an obvious cause for the lack of response is not readily identified. In addition, a product change may be required. The panel’s recommendations on choosing the right candidate and regimen for AIT are depicted in

In the UAE, the expert panel believes that using AIT is more cost-effective in the long term compared to other treatment options

## Discussion

These guidelines and recommendations were developed by a panel of UAE-based experts specifically for the management of AIT in patients with AR within the UAE healthcare system. They considered factors such as the local disease burden, available resources, and healthcare system structure. While these guidelines might offer valuable insights for healthcare professionals in other countries, it is acknowledged that they are not intended to be directly generalizable to other settings due to potential variations in local contexts. 

The panel provided valuable insights and recommendations for using AIT in managing AR within the UAE. They highlighted the use of AIT as a treatment option for patients whose AR symptoms are inadequately controlled by conventional methods and who demonstrate allergen sensitivity through allergy testing. Recently, many studies and guidelines reported safety and efficacy data that strongly support AIT as the only treatment that changes the natural course of AR and prevents exacerbation [51-55]. Real-world data on AIT efficacy and safety reinforces its long-term benefit in the management of AR patients [56]. Furthermore, considering the positioning of AIT in different international guidelines, the panel advocated that AIT may also be considered as a preventative measure by initiating it earlier in the disease course.

The panel underscored the importance of treatment duration of AIT, recommending a minimum of three years with the possibility of extending to five years for sustained clinical benefit. This recommendation is consistent with the latest European Academy of Allergy and Clinical Immunology (EAACI) guidelines on AIT for AR, which advocates for a minimum three-year treatment course to ensure long-term effectiveness [49,57]. Furthermore, a minimum one-year evaluation period recommendation aligns with the timeframe typically required to assess AIT efficacy [58].

In line with recent AIT guideline updates and real-world data, the expert panel recommended AIT as a disease-modifying treatment for patients with persistent symptoms despite optimal pharmacotherapy and avoidance measures. This aligns with long-term studies demonstrating AIT's effectiveness in improving disease control in such patients [49,52].

Consequently, the expert’s panel emphasized a stepwise approach to AR management, using intranasal corticosteroid nasal spray (INCS) as first-line therapy. These recommendations align with the Australian Society of Clinical Immunology and Allergy (ASCIA) and The BSACI guidelines, which recommend combination therapy with INCS plus intranasal antihistamines as first-line treatment [59,60]. In addition, the panel recommendations align with the International guidelines of the Korean Academy of Asthma, Allergy, and Clinical Immunology (KAAACI), which recommend INCS as the first-line treatment for moderate-to-severe or persistent AR [61]. Moreover, the expert’s panel emphasized the addition of saline nasal spray and progression to combination nasal sprays with steroids and antihistamines, or oral antihistamines, reflecting a measured approach based on symptom severity. While INCS is the established first-line treatment for moderate-to-severe AR according to international guidelines, a recent UAE consensus aligns with GARD guidelines in supporting the combination of INCS with an antihistamine as a first-line option for both SAR and PAR [62].

The panel's focus on SLIT reflects its growing popularity due to convenient administration, established efficacy, and favorable safety profile [63,64]. The panel highlighted the importance of accurate diagnosis (AR/rhinoconjunctivitis), documented treatment failure with standard medications (poor symptom control despite adequate pharmacotherapy), and confirmed sensitization (positive allergy tests) before considering AIT. This patient selection strategy aligns with established guidelines for AIT to encourage its use in patients for whom it would be of great need and benefit [1].

Recommendations produced in this manuscript are explicitly tailored to the UAE's healthcare system and environmental context, potentially limiting applicability to other countries in the Middle East/North Africa (MENA) region with different conditions and resources. Also, the study relies heavily on the opinions of the included group of experts, which may introduce subjective biases and limit generalizability. Additionally, the study focuses on specific AIT products available in the UAE, which may not include other options available globally, potentially limiting the applicability of the findings.

## Conclusions

The expert panel proposed recommendations for managing AR within the UAE, emphasizing the use of AIT in a selective population. This approach can potentially optimize clinical outcomes and improve cost-effectiveness within the UAE healthcare system by employing effective treatment strategies that prevent morbidity and additional use of healthcare resources. Ultimately, this consensus aims to improve care for AR patients in the UAE by providing healthcare professionals with expert guidance in managing this disease and using AIT in appropriately selected patients.
